# Supplementary material for: Mucoactive agent use in adult UK Critical Care Units: a survey of health care professionals’ perception, pharmacists’ description of practice, and point prevalence of mucoactive use in invasively mechanically ventilated patients
Source: PeerJ. 2020 May 4;8:e8828. doi: 10.7717/peerj.8828 (PMC7204825; doi:10.7717/peerj.8828)
Supplement: Supplemental Information 1 [file peerj-08-8828-s001.docx]

**Appendix**

Mucoactives in Critical Care Survey (MICCS) collaborators - Point prevalence survey

Aaisha Abdullahi, Guy's and St Thomas' NHS Foundation Trust; Adesola Olajitan, Ashford and St Peter's Hospitals NHS Foundation Trust; Aisling Corcoran, The Princess Alexandra Hospital NHS Trust; Alan Timmins , NHS Fife; Alastair Bovell, BUPA Cromwell Hospital; Alison O'Prey, NHS Greater Glasgow and Clyde; Allison Carruthers, NHS Borders; Amanda Varey, Royal United Hospitals Bath NHS Foundation Trust; Andrew Currie, Northern Health and Social Care Trust; Andrew Duncan, Blackpool Hospitals NHS Foundation Trust; Andrew Parsons, North Bristol NHS Trust; Anita Krishan, Brighton and Sussex University Hospitals NHS Trust; Anna Man, Basildon and Thurrock University Hospitals NHS Foundation Trust; Aoife Boyle, St George's University Hospitals NHS Foundation Trust; Bethan Thomas, Aberawe Bro Morgannwg University Health Board; Bill Wetherill, North Tees and Hartlepool NHS Foundation Trust; Breda Cronnolly, Buckinghamshire Healthcare NHS Trust; Bryan O'Farrell, Royal Free London NHS Foundation Trust; Camila Becker, Imperial College Healthcare NHS Trust; Carole Martin, NHS Lanarkshire; Charlotte Collins, Guy's and St Thomas' NHS Foundation Trust; Chloe Chan, Imperial College Healthcare NHS Trust; Chris Remmington, Royal Brompton and Harefield NHS Foundation Trust; Christie James, Oxford University Hospitals NHS Foundation Trust; Christine Turner, Calderdale and Huddersfield NHS Foundation Trust; Christopher Davey, George Eliot Hospital NHS Trust; Christopher Dixon, Manchester University NHS Foundation Trust; Christopher Meddings, Guy's and St Thomas' NHS Foundation Trust; Claire Hallett, Oxford University Hospitals NHS Foundation Trust; Clare Barton, Northumbria Healthcare NHS Foundation Trust; Clare Sales, Aintree University NHS Foundation Trust; Daniel Griffiths, University Hospitals Bristol NHS Foundation Trust; Daud Abdulla, Royal Free London NHS Foundation Trust; David Kean, Belfast Health and Social Care Trust; David Sapsford, Cambridge University Hospitals NHS Foundation Trust; Dawn Stewart, NHS Lanarkshire; Deborah Hughes, Wirral University Teaching Hospital NHS Foundation Trust; Deborah Webster, Barnsley Hospital NHS Foundation Trust; Dereck Gondongwe, University College Hospitals NHS Foundation Trust; Drilona Dobla, North Middlesex University Hospital NHS Trust; Egiehiokhin Obanor, St George's University Hospitals NHS Foundation Trust; Eleanor Martin, The Shrewsbury and Telford Hospital NHS Trust; Elena Mercer, Torbay and South Devon NHS Foundation Trust; Elizabeth Drewe, Leeds Teaching Hospitals NHS Trust; Emily Waterman, York Teaching Hospital NHS Foundation Trust; Emma Boxall, Salford Royal NHS Foundation Trust; Emma Goddard, Manchester University NHS Foundation Trust; Emma Graham-Clarke, Sandwell and West Birmingham Hospitals NHS Trust; Emma Tang, The Queen Elizabeth Hospital King's Lynn NHS Foundation Trust; Emma Watson, East Lancashire Hospitals NHS Trust; Erin Page, County Durham and Darlington NHS Foundation Trust; Fearn Davies, Oxford University Hospitals NHS Foundation Trust; Fiona Angus, The Christie NHS Foundation Trust; Franki Wilson, Leeds Teaching Hospitals NHS Trust; Fraser Hanks, Guy's and St Thomas' NHS Foundation Trust; Gillian Mulherron , The Newcastle upon Tyne Hospitals NHS Foundation Trust; Glen Cooper, University Hospitals Bristol NHS Foundation Trust; Greg Barton, St Helens and Knowsley Teaching Hospitals NHS Trust; Greg Musial , The Walton Centre NHS Foundation Trust; Gugandeep Samra, University Hospitals Birmingham NHS Foundation Trust; Gwenllian Pugh-Jones, Betsi Cadwaladr University Health Board; Harriet Hyman, Swansea Bay University Health Board; Heather Meldon, The Dudley Group NHS Foundation Trust; Heena Bhagat, Walsall Healthcare NHS Trust; Helen Barclay, NHS Lanarkshire; Helen Dillon, Norfolk and Norwich University Hospitals NHS Foundation Trust; Helen Leigh, Cardiff and Vale University Health Board; Helen March, The Pennine Acute Hospitals NHS Trust; Helen Stalker, Noble's Hospital Isle of Man; Holly Chapman, Oxford University Hospitals NHS Foundation Trust; Holly MacKenzie, The Newcastle upon Tyne Hospitals NHS Foundation Trust; Ian Rowlands, Barts Health NHS Trust; Jaimi Patel, Imperial College Healthcare NHS Trust; James Gall, NHS Grampian; James Gilmartin, Manchester University NHS Foundation Trust; Jane Wylie, NHS Highland; Jenna Thondee, Royal Free London NHS Foundation Trust; Jennifer Hughes, The Royal Liverpool and Broadgreen University Hospitals; Jennifer Murphy, NHS Lanarkshire; Jennifer Thomson, University Hospital Southampton NHS Foundation Trust; Jenny Oakley, University Hospitals of Morecambe Bay; Jess West, Brighton and Sussex University Hospitals NHS Trust; Jessica Hardwick-Smith, East Kent University Hospitals NHS Foundation Trust; Jignna Patel, West Hertfordshire Hospitals NHS Trust; Jill McDonald, Milton Keynes University Hospitals NHS Foundation Trust; Joanne Hanley, Northern Health and Social Care Trust; Joanne Peh, St George's University Hospitals NHS Foundation Trust; Joanne Wilkinson, North West Anglia NHS Foundation Trust; John Dade, Leeds Teaching Hospitals NHS Trust; John Warburton, University Hospitals Bristol NHS Foundation Trust; Jos Wickett, Countess of Chester Hospital MHS Foundation Trust; Joseph Tooley, Royal Surrey County Hospital NHS Foundation Trust; Joshua Harris, Airedale NHS Foundation Trust; Julie Bolger, Cardiff and Vale University Health Board; Karlie Williams, Cwm Taf Morgannwg University Health Board; Kate Leonardo, Wye Valley NHS Trust; Katharina Gernow, BMI The London Independent; Katherine Bell, The Newcastle upon Tyne Hospitals NHS Foundation Trust; Katherine Lee, Frimley Health NHS Foundation Trust; Katie Davies, Mid Cheshire Hospitals NHS Foundation Trust; Katie Hacking, Wrightington, Wigan and Leigh NHS Foundation Trust; Kevin Bazaz, University Hospitals of Leicester NHS Trust; Laura Granger, The Royal Bournemouth and Christchurch Hospitals NHS Foundation Trust; Laura Johnson, Frimley Health NHS Foundation Trust; Laura Radford, Cambridge University Hospitals NHS Foundation Trust; Laura Robinson, Northampton General Hospital NHS Trust; Lauren Nichol, Bolton NHS Foundation Trust; Lauren Woolsey, Stockport NHS Foundation Trust; Li Saw, The Royal Marsden NHS Foundation Trust; Linda Barrow, Royal Papworth Hospital NHS Foundation Trust; Lisa Higgins, University Hospitals of North Midlands NHS Trust; Lorna Morrow, Western Health and Social Care Trust; Louise Greenwell, County Durham and Darlington NHS Foundation Trust; Lucy Holmes, North Bristol NHS Trust; Lydia Wang, BMI Priory; Lyndsey Young, The Newcastle upon Tyne Hospitals NHS Foundation Trust; Lynne Hamilton, University Hospitals of Morecambe Bay NHS Foundation Trust; Mark Graham, South Tyneside and Sunderland NHS Foundation Trust; Mark Shortland, Dorset County Hospital NHS Foundation Trust; Martyn Gall, Golden Jubilee National Hospital NHS Scotland; Matthew Seymour, Brighton and Sussex University Hospitals NHS Trust; Matthew Walton, South Tyneside and Sunderland NHS Foundation Trust; Matthew Youngman, West Suffolk NHS Foundation Trust; Maxine Robinson, Liverpool Heart and Chest Hospital NHS Foundation Trust; Mayhul Patel, Southend University Hospital NHS Foundation Trust; Meera Dalal, University College London Hospitals NHS Foundation Trust; Melissa Nankoo, Barts Health NHS Trust; Michael Bowe, Gateshead Health NHS Foundation Trust; Mike Barker, Guy's and St Thomas' NHS Foundation Trust; MingHooi Gan, Lancashire Teaching Hospitals NHS Foundation Trust; Miriam Formica, Whittington Health NHS Trust; Morna Ball, NHS Greater Glasgow and Clyde; Natasha Hamilton, Buckinghamshire Healthcare NHS Trust; Nerys Winnard, Chesterfield Royal Hospital NHS Foundation Trust; Nisha Bhudia, Royal Brompton and Harefield NHS Foundation Trust; Nishma Gadher, University College London Hospitals NHS Foundation Trust; Odran Farrell, University Hospitals Birmingham NHS Foundation Trust; Olivia Kanka, Cambridge University Hospitals NHS Foundation Trust; Parul Sudra, Croydon Health Services NHS Trust; Patricia Chan, East Kent Hospitals University NHS Foundation Trust; Patricia Crossey, Royal Liverpool and Broadgreen University Hospitals NHS Trust; Peter Buckner, NHS Tayside; Peter Gray, University Hospitals Plymouth NHS Trust; Punam Mehra, BMI The Clementine Churchill; Rachel Llewellyn, Gloucestershire Hospitals NHS Foundation Trust; Rachel Whitaker, Sheffield Teaching Hospitals NHS Foundation Trust; Rani Ahmed, Barking, Havering and Redbridge University Hospitals NHS Trust; Rebecca Tarrant, East Kent Hospitals University NHS Foundation Trust; Rhona Sloss, King's College Hospital NHS Foundation Trust; Ricardo Pinheiro, BMI The Park; Richard Gant, East Suffolk and North Essex NHS Foundation Trust; Richard Ladner, Barts Health NHS Trust; Rikesh Patel, Homerton University Hospital NHS Foundation Trust; Rukhsana Mahmood, BMI Alexandra; Ruth Forrest, NHS Greater Glasgow and Clyde; Sabiha Ravat, Calderdale and Huddersfield NHS Foundation Trust; Salma Al-Maskari, University Hospitals of Leicester NHS Trust; Sam Heard, Royal Papworth Hospital NHS Foundation Trust; Sam Walters, The Royal Wolverhampton NHS Trust; Samantha Dickinson, Lewisham and Greenwich NHS Trust; Samantha Dymond, Northern Devon Healthcare NHS Trust; Samantha Emery, Sheffield Teaching Hospitals NHS Foundation Trust; Samantha Stevens, Royal Cornwall Hospitals NHS Trust; Sandra Sowah, Medway NHS Foundation Trust; Sarah Boulger, The Pennine Acute Hospitals NHS Trust; Sarah Connelly, NHS Lanarkshire; Sarah Connop, Brighton and Sussex University Hospitals NHS Trust; Sarah Karthauser, Weston Area Health NHS Trust; Sarah Khorshid, Guy's and St Thomas' Hospitals NHS Foundation Trust; Sarah Kimpton, The Rotherham NHS Foundation Trust; Sarah Sawieres, King's College Hospital NHS Foundation Trust; Sarb Sarai, East and North Hertfordshire NHS Trust; Shaneeza Jamil, Brighton and Sussex University Hospitals NHS Trust; Sharanjit Kaur, University Hospitals of Leicester; Shu Yi Tan, King's College Hospital NHS Foundation Trust; Shumaila Asif, North Cumbria University Hospitals NHS Trust; Sinead O'Halloran, Lewisham and Greenwich NHS Trust; Sinead Tynan, Barking, Havering and Redbridge University Hospitals NHS Trust; Sonia Moore, Epsom and St Helier University Hospitals NHS Trust; Steven Lockwood, South Tees Hospitals NHS Foundation Trust; Susan Lee, Luton and Dunstable University Hospital NHS Foundation Trust; Susan Muirden, BMI The Hampshire; Susan Ramsay, Barts Health NHS Trust; Taiya Large, Bedford Hospital NHS Trust; Tariq Azamgarhi, The Royal National Orthopaedic Hospital NHS Foundation Trust; Vicky Glaister, University Hospitals Coventry and Warwickshire NHS Trust; Vikki Loyd, University Hospitals of North Midlands NHS Trust; Vimbayi Mushayi, Western Sussex Hospitals NHS Foundation Trust; Wendy Lawson, East Cheshire NHS Trust; Ya-Hui Liang, University Hospitals Plymouth NHS Trust; Yin Yang Pang, Manchester University NHS Foundation Trust
