# Supplementary material for: Mucoactive agent use in adult UK Critical Care Units: a survey of health care professionals’ perception, pharmacists’ description of practice, and point prevalence of mucoactive use in invasively mechanically ventilated patients
Source: PeerJ. 2020 May 4;8:e8828. doi: 10.7717/peerj.8828 (PMC7204825; doi:10.7717/peerj.8828)
Supplement: Supplemental Information 3 [file peerj-08-8828-s003.docx]

Questionnaire for practitioner level survey

Survey of mucoactive use in UK adult critical care units - survey of personal practice

Q1: Please select the type of your unit (please take this survey only if you work in an adult critical unit, includes ICU/HDU/HMU).

Mixed medical/surgical critical care unit (including trauma)

Medical critical care unit

Surgical critical care unit (including trauma)

Neuro critical care unit

Cardiac critical care unit (including cardiac surgery)

Liver critical care unit

Other (please specify)

Q2: Please select the professional group that you belong to.

Medical – Consultant

Medical – Trainee

Medical – Other

Physiotherapy

Nursing

Q3: Are you a member of The Intensive Care Society UK

Yes

No

Q4: When do you most commonly use mucoactive agents in mechanically ventilated patients? Please enter details on your personal practice and not your unit practice. Please rank one or more categories as necessary, where 1 is the most common, 2 is the next most common, etc (We are not currently seeking views on the use of mucoactives solely to obtain sputum samples). The following options are not listed in any particular order. If you rank "We don't use mucoactive agents", you should not rank any of the other choices.

COPD

ARDS

Pneumonia

Bronchiectasis

“Weak Cough”

“Thick Secretions”

Routine in most invasively ventilated patients

I don’t use mucoactive agents

Other

Q5: If mucoactives are used for thick secretions, what criteria is used to classify sputum viscosity?

Clinical observation only (by doctor, nurse or physio)

Established criteria for sputum viscosity

Q6: In your opinion, what are the expected clinical benefit(s) of using mucoactive agents in patients that would influence your clinical practice? Please rank one or more answers  to reflect the main expected benefits, where 1 is the foremost benefit, 2 is the next foremost benefit, etc. You do not have to rank all options. The following outcomes are not listed in any particular order. If you rank "There are no benefits" you should not rank any of the other choices.

Reduced time of mechanical ventilation

Reduced reintubation rate

Reduced ICU length of stay

Reduced hospital length of stay

Reduced mortality

Improved gas exchange

Sputum clearance

There are no benefits

Q7: Do you regularly use the following as mucoactive agents either nebulised or directly flushed down the endotracheal tube / tracheostomy in patients with acute respiratory failure (exclude use to obtain sputum samples). Choose as many as applicable. Please enter details on your personal practice and not the unit practice.

Isotonic sodium chloride

Hypertonic saline

N-Acetylcisteine

Dornase alpha/DNase

If Other, please specify medication and dose

Q8: What type of nebuliser(s) do you use in your unit in mechanically ventilated patients? If you have access to more than one nebuliser, rank the most common as 1 and the second most common as 2 etc. If you have only one type, rank it as 1 and do not rank others.

Jet nebuliser

Ultrasonic

Vibrating mesh

Q9: Do you use systemically delivered (orally, enterally or parenterally) mucoactive agents in mechanically ventilated patients with acute respiratory failure? (exclude use to obtain sputum samples).

N-Acetylcisteine

Carbocisteine

Other (please specify medication and dose)

Q10: Mucoactive agents are widely used in critical care without clear evidence of benefit or harm. Regardless of your view as to whether mucoactives are beneficial or not, how important do you think it is that we answer the following research question: “Does the use of either inhaled or systemic mucoactives in mechanically ventilated or non-ventilated patients with acute respiratory failure improve patient outcomes?"

Very important

Important

Uncertain

Unimportant

Not at all important

Q11: Would you or your unit be interested in participating in a research study of mucoactive agents in mechanically ventilated patients with acute respiratory failure?

Yes

No

Q12: If your answer to the above question is yes, one of the intervention arms will be "no mucoactive agents". Would you or your unit still consider participating in this study?

Yes

No

Q13: If the answer to question 13 is yes, we would like to know if you or your unit would still consider participating in this study if the use of isotonic saline (0.9% saline) nebulisers is disallowed.

Yes

No

Q14: If the answer to question 13 is yes, we would like to know if you or your unit would still consider participating in this study if the use of isotonic saline (0.9% saline) nebulisers is disallowed.

Yes

No

Q15: If the answer to question 13 is yes, would you randomise to a factorial study design investigating the most commonly used oral mucoactive agent and inhaled mucoactive agent.

Yes

No
