# Supplementary material for: Mucoactive agent use in adult UK Critical Care Units: a survey of health care professionals’ perception, pharmacists’ description of practice, and point prevalence of mucoactive use in invasively mechanically ventilated patients
Source: PeerJ. 2020 May 4;8:e8828. doi: 10.7717/peerj.8828 (PMC7204825; doi:10.7717/peerj.8828)
Supplement: Supplemental Information 4 [file peerj-08-8828-s004.docx]

**Unit level survey questions – Text from Survey Monkey**

Please provide some contact details - these are only required in case of a query or where there is need for clarification. These details will not be passed to third parties

*The first two fields require an entry

Name*

Email Address*

Phone Number

_____________

Please select the unit you are responding for. The answer list has been derived from participant lists in ICNARC-CMP July 2017 (England, Wales, Northern Ireland) and SICSAG December 2016 (Scotland). Some critical care services are characterised by more than one designated unit, i.e. there could be a specific line for an HDU and a separate line for an ICU rather than a combined line for HDU/ICU. The actual designation in the list in this survey is determined by coding in ICNARC and SICSAG. If you can answer for more than one unit, please complete one survey response for each location you can answer for

(Format: Country, Overarching Network or Country, Hospital name, Unit name)

Not - Listed (please select this option and add name manually in text box below)

_____________

When do you most commonly use mucoactive agents in patients on the unit?

Please rank one or more categories as necessary to reflect practice on the unit, where 1 is the most common, 2 is the next most common, etc (We are not currently seeking views on the use of mucoactives solely to obtain sputum samples, or in Cystic Fibrosis or ECMO). The following list order is randomised {randomise functionality used – list order randomised for each respondent}

- COPD
- ARDS
- Pneumonia
- Bronchiectasis
- "Weak Cough"
- "Thick Secretions"
- Routine in most invasively ventilated patients
- We don't use mucoactive agents
- Routine in most non-invasively ventilated patients

_____________

Are there any other commonly used indications for mucoactive agents on [unit name from Qu2]? (We are not currently interested in the use of mucoactives solely to obtain sputum samples, or in Cystic Fibrosis or ECMO)(This question does not require an answer)

[Text Box]

_____________

What are the expected clinical benefit(s) of using mucoactive agents in patients on your unit? We are aware that improved sputum clearance is a likely desired option, but we are more interested in the other measurable outcomes listed. Please rank one or more answers as necessary to reflect the main expected benefits, where 1 is the foremost benefit, 2 is the next foremost benefit, etc. You do not have to rank all options. The following list order is randomised {randomise functionality used – list order randomised for each respondent}

- Reduced time on mechanical ventilation
- Reduced intubation / reintubation rate
- Reduced ICU length of stay
- Reduced hospital length of stay
- Reduced mortality
- Improved gas exchange
- There are no benefits

_____________

Does your unit use: Topically delivered Isotonic Sodium Chloride 0.9% (nebulised or directly flushed down the endotracheal tube / tracheostomy) as a mucoactive agent in patients with acute respiratory failure? (exclude use to obtain sputum samples, use in Cystic Fibrosis and use in ECMO)

[Yes] [No]

_____________

Does your unit use: Topically delivered Hypertonic Sodium Chloride (nebulised or directly flushed down the endotracheal tube / tracheostomy) as a mucoactive agent in patients with acute respiratory failure and if so, what is the most commonly used concentration? (exclude use to obtain sputum samples, use in Cystic Fibrosis and use in ECMO)

[Yes] [No] [Text box]

_____________

Does your unit use: Topically delivered N-Acetylcysteine (nebulised or directly flushed down the endotracheal tube / tracheostomy) as a mucoactive agent in patients with acute respiratory failure and if so, what is the most commonly used concentration? (exclude use to obtain sputum samples, use in Cystic Fibrosis and use in ECMO)

[Yes] [No] [Text box]

_____________

Does your unit use: Topically delivered dornase alfa / DNase / Pulmozyme (nebulised or directly flushed down the endotracheal tube / tracheostomy) as a mucoactive agent in patients with acute respiratory failure? (exclude use to obtain sputum samples, use in Cystic Fibrosis and use in ECMO)

[Yes] [No]

_____________

Does your unit use: Any other medication topically (nebulised or directly flushed down the endotracheal tube / tracheostomy) as a mucoactive agent in patients with acute respiratory failure? (exclude use to obtain sputum samples, use in Cystic Fibrosis and use in ECMO)(You can skip this question if there are none)

[Text box]

_____________

Does your unit use: Systemically delivered N-acetylcysteine (Orally, enterally or parenterally) as a mucoactive agent in patients with acute respiratory failure? (exclude use to obtain sputum samples, use in Cystic Fibrosis and use in ECMO)

[Yes] [No]

_____________

Does your unit use: Systemically delivered Carbocysteine (Orally, enterally) as a mucoactive agent in patients with acute respiratory failure? (exclude use to obtain sputum samples, use in Cystic Fibrosis and use in ECMO)

[Yes] [No]

_____________

Does your unit use: Any other medication systemically delivered (orally, enterally or parenterally) as a mucoactive agent as a mucoactive agent in patients with acute respiratory failure? (exclude use to obtain sputum samples, use in Cystic Fibrosis and use in ECMO)(you can skip this question if there are none)

[Yes] [No]

_____________

If your unit has a policy/protocol/guideline for the use of mucoactives, please indicate the title in the text box below and email the document to MiCCs2018@protonmail.com with the subject title "MUCOACTIVE SURVEY" Please provide references/citations for any protocol (if not already included within it)

_____________

If there is no unit policy or protocol, please provide references/citations upon which unit practice is based

[Text Box]

_____________

Mucoactive agents are widely used in critical care without clear evidence of benefit or harm. Regardless of your view as to whether mucoactives are beneficial or not, how important do you think it is that we answer the following research question:

“Does the use of either inhaled or systemic mucoactives in mechanically ventilated patients with acute respiratory failure improve patient outcomes?”

[Very important] [Important] [Uncertain] [Unimportant] [Not at all important]

_____________

Would you or your unit be interested in participating in a research study of mucoactive agents in mechanically ventilated patients with acute respiratory failure?

[Yes] [No]
